# Current management of atrial fibrillation in routine practice according to the last ESC guidelines: an EHRA physician survey—how are we dealing with controversial approaches?

**DOI:** 10.1093/europace/euae012

**Published:** 2024-01-16

**Authors:** Jose M Guerra, Zoraida Moreno Weidmann, Laura Perrotta, Arian Sultan, Ante Anic, Andreas Metzner, Rui Providencia, Serge Boveda, Julian Chun

**Affiliations:** Department of Cardiology, Hospital de la Santa Creu i Sant Pau, IR SANT PAU, Universitat Autònoma de Barcelona, CIBERCV, Sant Antoni M. Claret 167, 08025 Barcelona, Spain; Department of Cardiology, Hospital de la Santa Creu i Sant Pau, IR SANT PAU, Universitat Autònoma de Barcelona, CIBERCV, Sant Antoni M. Claret 167, 08025 Barcelona, Spain; Arrhythmia Unit, Careggi University Hospital, Florence, Italy; Department of Electrophysiology, Heart Center, University of Cologne, Köln, Germany; Department for Cardiovascular Diseases, University Hospital Centre, Split, Croatia; Department of Cardiology, University Heart and Vascular Center Hamburg, University Hospital Hamburg-Eppendorf, Hamburg, Germany; Electrophysiology Department, Barts Heart Centre, St. Bartholomew’s Hospital, London, UK; Institute of Health Informatics Research, University College London, London, UK; Department of Cardiology, Heart Rhythm Management, Clinique Pasteur, Toulouse, France; Brussels University VUB, Brussels, Belgium; Cardioangiologisches Centrum Bethanien (CCB), Medizinische Klinik III, Agaplesion Markus Krankenhaus, Frankfurt am Main, Germany

**Keywords:** Atrial fibrillation, Guidelines, Clinical practice, Comorbidities, Anticoagulation, Antiarrhythmic treatment, Ablation

## Abstract

**Aims:**

Although guidelines for the management of atrial fibrillation (AF) are regularly published, many controversial issues remain, limiting their implementation. We aim to describe current clinical practice among European Heart Rhythm Association (EHRA) community according to last guidelines.

**Methods and results:**

A 30 multiple-choice questionnaire covering the most controversial topics related to AF management was distributed through the EHRA Research Network, National Societies, and social media between January and February 2023. One hundred and eighty-one physicians responded the survey, 61% from university hospitals. Atrial fibrillation screening in high-risk patients is regularly performed by 57%. Only 42% has access to at least one programme aiming at diagnosing/managing comorbidities and lifestyle modifications, with marked heterogeneity between countries. Direct oral anticoagulants are the preferred antithrombotic (97%). Rhythm control is the preferred strategy in most AF phenotypes: symptomatic vs. asymptomatic paroxysmal AF (97% vs. 77%), low vs. high risk for recurrence persistent AF (90% vs. 72%), and permanent AF (20%). I-C drugs and amiodarone are preferred while dronedarone and sotalol barely used. Ablation is the first-line therapy for symptomatic paroxysmal AF (69%) and persistent AF with markers of atrial disease (57%) and is performed independently of symptoms by 15%. In persistent AF, 68% performs only pulmonary vein isolation and 32% also additional lesions.

**Conclusion:**

There is marked heterogeneity in AF management and limited accordance to last guidelines in the EHRA community. Most of the discrepancies are related to the main controversial issues, such as those related to AF screening, management of comorbidities, pharmacological treatment, and ablation strategy.

## Graphical Abstract

**Graphical Abstract:**
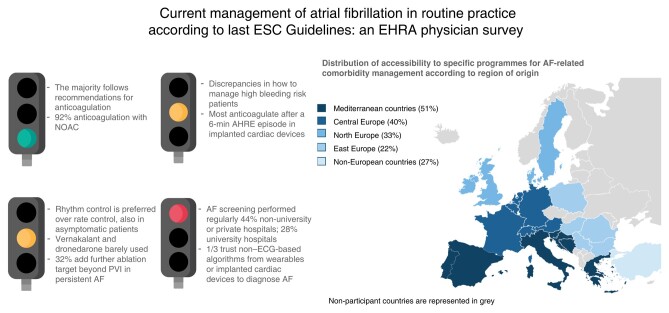

**What’s new?**

- This European Heart Rhythm Association (EHRA) survey explores current clinical practice in atrial fibrillation (AF) diagnosis and management among the EHRA community and accordance to the recent 2020 ESC Guidelines.
- The survey shows marked heterogeneity in clinical practice and limited accordance to the last ESC guidelines, mostly for the main controversial issues related to AF management.
- The survey also shows a marked heterogeneity in accordance to guidelines based on geographical origin and health care system.
- The areas with major discrepancies are those related to AF screening and interpretation of non–ECG-based monitoring tools, comorbidity management, pharmacological treatment, and ablation strategy.

## Introduction

Atrial fibrillation (AF) is one of the main current areas of interest in clinical and interventional electrophysiology. Clinical guidelines for the management of AF are regularly issued, and their content keeps changing as knowledge increases.^1,2^ However, many controversial issues and gaps in knowledge remain, hampering its diffusion and implementation and resulting in a marked heterogeneity in the management of patients.^3,4^

This survey was developed to describe the current clinical practice in AF diagnosis and management among the European Heart Rhythm Association (EHRA) community and its accordance to the recent 2020 ESC Guidelines.^1^ Special attention was paid to the most controversial topics related to management and the new strategies recently proposed. The aim of the survey was to detect the main areas of uncertainties and discrepancies with guideline recommendations and the patterns that determine these differences.

## Methods

An online questionnaire consisting of 30 multiple-choice questions (see Supplementary material) was sent to the EHRA Research Network. Invitations to participate were also distributed through the National Societies and via social media platforms. This physician-based EHRA survey was conducted between January and February 2023. To detect different management patterns, respondents are classified according to the European region from which they come, the health care system (Beveridge vs. Bismarck) of the country, and the type of hospital they are working in (university hospital, private or specialized hospital, and non-university hospital). Categorical variables are presented numerically with absolute percentages (%). Data were analysed using descriptive statistical methods.

## Results

### Demographics

The questionnaire was answered by a total of 181 participants from 22 European countries and 14 non-European countries belonging to the EHRA community (see Supplementary material online, *Table*).

Most of the participants worked at university hospitals (112, 61%), followed by non-university hospitals (36, 20%), private centres (16, 9%), and specialized cardiac centres (15, 8%). Years of experience treating AF patients of the responders was 15 ± 9 years. Medical resources related to AF management recommended by the Guidelines^1^ available at the centres are listed in *Table 1*. Only 65 participants (36%) reported to have access to all the resources at their centres.

**Table 1:** Medical resources related to AF management recommended by the guidelines available at the centres where the participants worked

Medical service	Number	Percentage
Outpatient cardiology	160	88
Cardiology ward	156	86
Electrophysiology department	165	91
AF ablation	156	86
Coagulation department	97	53
Device implantation/follow-up	165	91
Left atrial appendage closure	120	66
Cardiac surgery	103	57

AF, atrial fibrillation.

University hospitals and specialized cardiac centres performed more frequently AF ablations (101, 91% and 13, 87%, respectively), compared to private centres (14, 82%) and non-university hospitals (27, 75%). Overall, the centres where 103 of the responders worked (57%) were tertiary care facilities.

A total of 75% of the responders were identified as the main cardiologist responsible for the holistic diagnosis and management of AF while the remaining 25% of the responders were only involved in performing invasive procedures.

In those centres where outpatient follow-up visits are carried out (159, 88%), the physicians have an average of 65 ± 53 (median 50, interquartile range [IQR] 75) patients per month. In those centres performing ablation procedures (156, 86%), physicians perform a mean of 18 ± 14 AF (median 15, IQR 17) procedures per month, while those performing left atrial appendage (LAA) closure procedures (120, 66%), physicians have a mean of 3 ± 7 per month (median 1, IQR 2).

### Atrial fibrillation opportunistic screening

A total of 61 (33%) physicians report performing regularly opportunistic or systematic screening for AF in high-risk patients (defined by the current guidelines as those aged over 65 years and/or with other cardiovascular risk factors),^1^ and 44 (24%) of the physicians do not perform the screening by themselves but rather by other colleagues in the same area of reference. In contrast, 45 (25%) perform such screening only occasionally and 30 (16%) never or rarely. Participants from East European and non-European countries (56 and 55%), followed by Mediterranean European participants (34%), perform more frequently opportunistic AF screening compared to Central and North European participants (21 and 0%).

The preferred method for AF screening is a rhythm strip or 12-lead ECG performed during consultation, as reported by 152 physicians (83%), followed by Holter monitoring (127 physicians, 69%), by wearable devices (110,60%), by pulse taking or auscultation (98, 54%), and a minority (4%) by implantable loop recorders (ILRs) or implanted cardiac devices (ICDs). In the interpretation of automatic AF diagnostic algorithms generated by wearables or cardiac implantable devices (CIDs), 103 physicians (56%) establish the diagnosis of AF only if clear tracings are available and the arrhythmia is well recognizable. Meanwhile, 50 physicians (27%) trust AF episodes, including those without tracings [based on photoplethysmography-based tracings or atrial high-rate episodes (AHREs)]. A smaller number of physicians (17, 9%) establish the diagnosis of AF only after confirmation by ECG or Holter monitoring. Another 4% (eight physicians) do not incorporate or do not trust such information for the diagnosis of AF.

### Comorbidity screening and management

A 42% of the participants have access to at least one specific programme aimed at diagnosing and managing comorbidities and lifestyle modifications (*Table 2*), primarily targeting obstructive sleep apnoea, and only 6% has access to multifactorial and multidisciplinary programmes to manage all related comorbidities. Furthermore, 37% of the participants have no access to any such programmes and attempt to manage it by themselves during the cardiac consultation. In contrast, 21% of participants are not involved in the management of comorbidities and lifestyle modifications as part of their AF treatment.

**Table 2:** Availability of specific programmes aimed at diagnosing and managing comorbidities and lifestyle modifications in AF patients

Type of programme	Number	Percentage
OSAS screening and treatment	66	36
Education programme by nurses	37	20
Nutritionist/endocrinologist	33	18
Exercise programme	39	21
Hypertension unit	3	2

OSAS, obstructive sleep apnoea syndrome.

**Figure 1:**
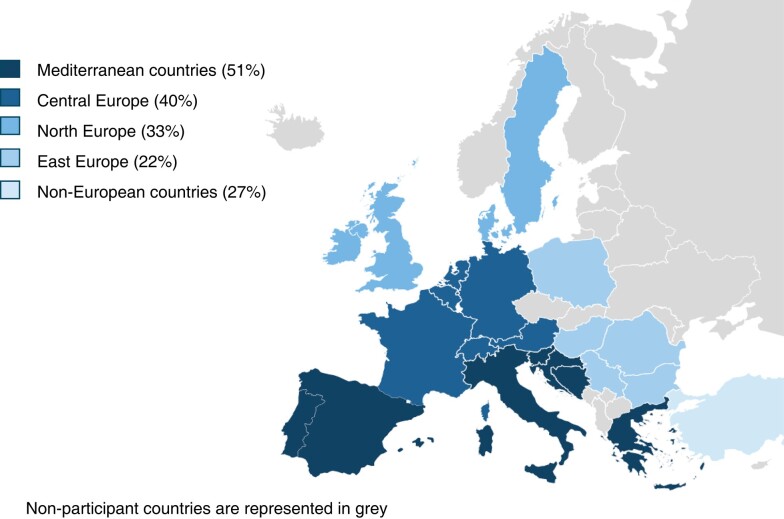
Accessibility to specific programmes for AF-related comorbidity management according to region of origin. Percentage of participants that have access to at least one specific programme as per guideline recommendation is represented. AF, atrial fibrillation.

A marked heterogeneity is observed when considering the geographical origin of the responder. Mediterranean and Central European countries have better access to specific programmes for the diagnosis and management of comorbidities and lifestyle modifications (51 and 40%, respectively), as opposite to North European, East European, and non-European countries (33, 22, and 27%, respectively; *Figure 1*). Additionally, 45% of participants consider deferring the decision for AF ablation after controlling the comorbidities or unhealthy lifestyles.

### Stroke prevention—anticoagulation

Most of the participants (168, 92%) prescribe non-vitamin K oral anticoagulants (NOACs) as first-line anticoagulant. Only six participants (3%) use them in cases of low time in therapeutic range under vitamin K antagonists (AVK) and six participants (3%) use AVK as first-line therapy. In low-risk patients for thromboembolic events, defined as CHA_2_DS_2_-VASc score of 1 in men or 2 in women, the decision to initiate chronic anticoagulation engages shared decision-making with the patient for 81 physicians (44%), it is directly recommended by 58 physicians (32%) and 28 physicians (15%) base their recommendation on the relative weight of each risk factor. A minority does not recommend any treatment (7, 4%) or prescribes antiplatelet therapy (6, 3%). No differences were found according to the health care system. Notably, in northern European countries, all participants discuss the therapeutic options with patients, while half of Mediterranean and Central European participants do so, and non-European participants are more likely to directly recommend starting anticoagulation.

**Figure 2:**
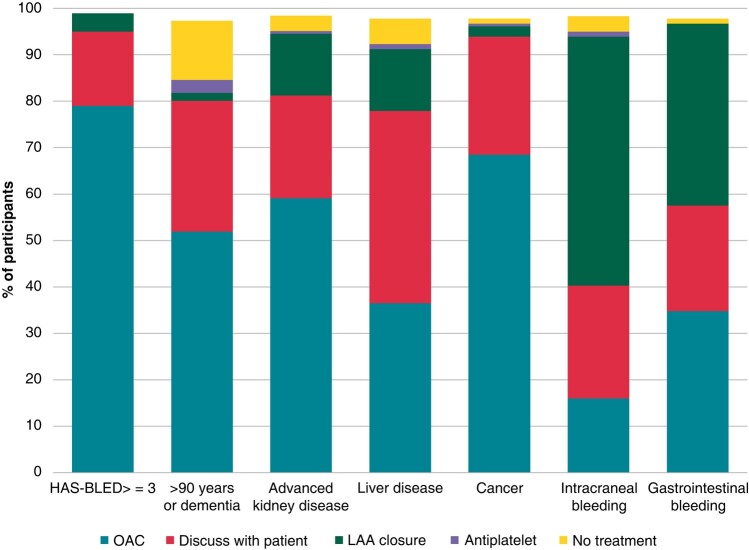
Preferred anticoagulation strategy for high bleeding risk AF patients. LAA, left atrial appendage; OAC, oral anticoagulation.

The preferred anticoagulation strategy for high bleeding risk patients is represented in *Figure 2*. Most of the participants recommend starting anticoagulation in patients with high HAS-BLED score, advanced chronic kidney disease, and cancer. Meanwhile, LAA closure is the most preferred approach for intracranial (53%) or gastrointestinal bleeding (39%) while it is marginally prescribed in other situations (2% in dementia to 13% in liver and kidney disease). About a quarter of the physicians discuss the approach with the patient in all scenarios, except in the case of liver failure, where up to 40% of the physicians prefer to discuss this question.

Regarding self-limited post-operative AF after non-cardiac surgery, 91 physicians (50%) recommend long-term anticoagulation in high CHA_2_DS_2_-VASc patients, 42 physicians (23%) recommend it during 3 months after the AF episode, 7 physicians (15%) would prefer shared decision-making with the patient, and 19 physicians (10%) rarely recommend anticoagulation.

**Figure 3:**
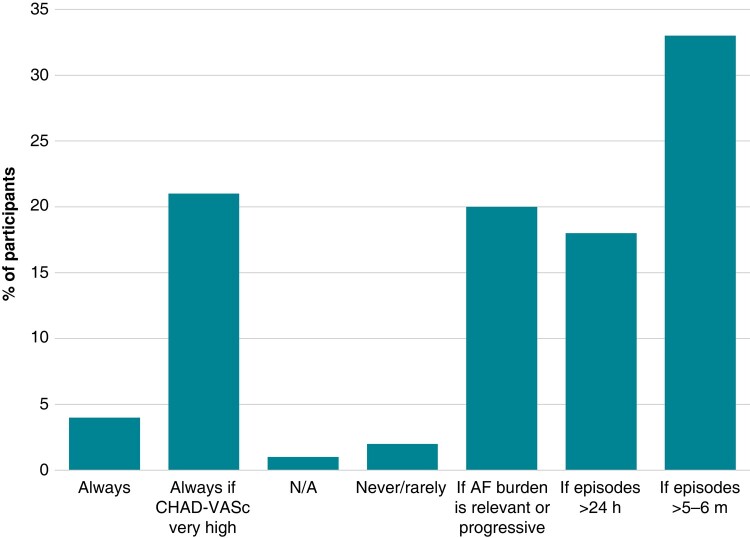
Participants’ decision on anticoagulation for patients with AHRE registered in cardiac implantable devices and elevated CHA_2_DS_2_-VASc score. AHRE, atrial high-rate episode. AF, atrial fibrillation; N/A, data not provided.

In the case of AF episodes detected by CID with high CHA_2_DS_2_-VASc score, most of the responders take AF duration into account when considering anticoagulation: 61 physicians (33%) recommend it when a duration of the episodes recorded by the system is longer than 5–6 min, while 32 physicians (18%) only if the episodes last >24 h. Thirty-six physicians (20%) recommend the initiation of anticoagulation if the AF burden is relevant or a progression in the episodes duration is detected. The rest of the participants base its decision on the thromboembolic risk: 38 physicians (21%) always start anticoagulation therapy if the score is very high, independently of the duration of episodes or AF burden, 8 physicians (4%) always independently of the CHA_2_DS_2_-VASc score, and 3 physicians (2%) never or rarely (*Figure 3*).

### Rhythm vs. rate control

Rhythm control is the preferred strategy in most AF phenotypes: in paroxysmal AF, if symptomatic by 177 physicians (97%), falling to 141 physicians (77%) if asymptomatic; in persistent AF, in low risk for recurrence by 164 participants (90%), falling to 132 participants (72%) in patients with risk for recurrence, defined as per guidelines as those with significantly enlarged left atrial volume, advanced age, long AF duration, renal dysfunction, or other cardiovascular risk factors.^1^ Additionally, rhythm control is also the preferred strategy (173 participants, 95%) for persistent AF in patients with low ejection fraction (EF). Notably, for 36 physicians (20%), rhythm control strategy is still the chosen approach for permanent AF, challenging its own definition.

**Figure 4:**
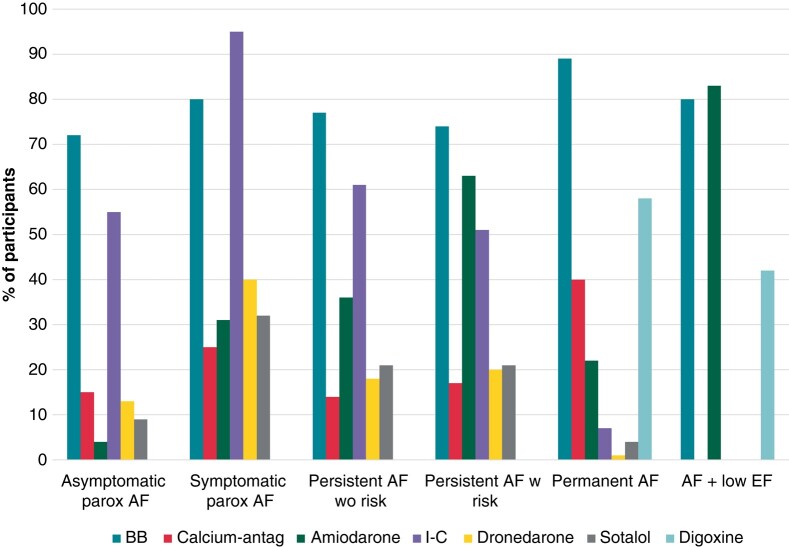
Use of antiarrhythmic drugs as per AF phenotype. AF, atrial fibrillation; BB, beta-blockers; Calcium-antag, calcium-antagonists, EF, ejection fraction; parox, paroxysmal; w, with; wo, without.

The antiarrhythmic drugs (AADs) most frequently prescribed, independently of the AF phenotype, are beta-blockers. Drugs of the I-C group are preferred for paroxysmal and persistent AF with low risk of recurrence, while amiodarone was preferred for persistent AF with higher risk of recurrences, as well as for reduced EF. Dronedarone and sotalol are barely used and mostly in paroxysmal symptomatic AF (*Figure 4*). Regarding dronedarone, it is never or rarely used by most of the physicians (96, 53%). Its major indication is contraindication for amiodarone (48, 26%), intolerance to other AADs (44, 24%), or failure of other AADs (31, 17%). Only 12 physicians (7%) choose it as first-line therapy.

Pharmacological cardioversion with vernakalant is a first option therapy for 94 physicians (52%) and a second option therapy (after failure of electrical cardioversion or with other drugs) for 31 physicians (17%). Forty-six physicians (23%) have no access at their institution or use it barely despite being available. No remarkable regional differences are observed.

### Ablation

Ablation is the preferred first-line therapy for symptomatic paroxysmal AF (69%), persistent AF with low risk of recurrence (57%), and persistent AF with associated low EF (74%). Ablation is considered as second-line therapy (after failure of electrical cardioversion or AADs) for asymptomatic paroxysmal AF (54%), persistent AF with risk of recurrence (53%), and long-standing persistent AF (46%).

Atrial fibrillation ablation would never be recommended in permanent AF by 41%, in asymptomatic patients by 23%, persistent AF with risk of recurrence by 4%, and persistent AF with low EF by 2%.

In paroxysmal AF, 171 physicians (94%) perform only pulmonary vein isolation as the index strategy, while in persistent AF, 59 physicians (32%) perform additional lesions beyond pulmonary vein isolation (PVI).

Cavo-tricuspid isthmus (CTI) ablation during the same procedure of AF ablation is performed by 81 physicians (45%), only if the patient had a previous history of documented typical flutter on ECG, and by 64 physicians (35%), if typical flutter is previously documented or is induced during the procedure. Twenty physicians (11%) never or rarely perform CTI ablation during the same procedure. A minority of seven physicians (4%) perform it systematically.

**Figure 5:**
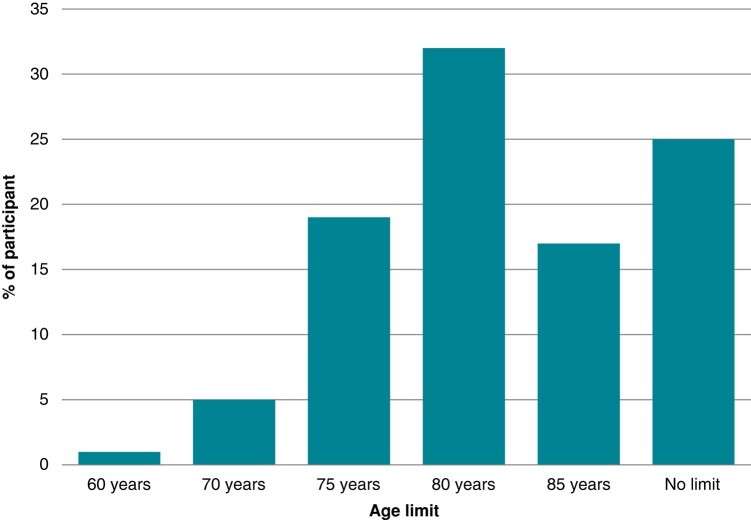
Age limit for AF ablation assumed by the participant physicians. AF, atrial fibrillation.

The most frequent factor that influences the decision for performing AF ablation is patient’s decision (167 physicians, 92%), followed by patients’ comorbidities (165 physicians, 89%), LA remodelling (154 physicians, 86%), and AF phenotype (146 physicians, 81%). The presence of symptoms is considered by 155 physicians (85%). Remarkably, age is the least important factor to decide for ablation (78%). Age limits for ablation assumed by the participants are represented in *Figure 5*.

Pace and ablate is frequently performed as last-line therapy after ablation in cases of no symptom relief or in cases where heart rate is difficult to control (77%), followed by 38% of participants that would recommend it if no benefit from ablation is expected. Eleven per cent performs it when ablation fails, independently of symptoms or heart rate, 6% rarely and 2% frequently, and independently of the ablation status.

## Discussion

This EHRA survey has found numerous discrepancies between guideline recommendations^1^ and clinical practice in all explored fields. The most controversial areas are those related to AF screening and interpretation of non–ECG-based monitoring tools, comorbidity management, ablation strategy, and pharmacological treatment.

### Atrial fibrillation screening

Guidelines recommend performing screening to detect AF opportunistically in patients ≥65years (Class I) and systematically in individuals ≥75 years or in those at high risk of stroke (Class IIa).^1^ The survey shows that regular screening is not broadly performed. Higher screening levels were observed in responders working at non-university hospitals and private/specialized cardiology centres (42 and 44%, respectively). Remarkably, only 28% of the physicians from university hospitals reported to perform it, although 19% recognizes that it is made in their reference area. This finding may reflect a lack of time and resources destinated to preventive politics or a lack of awareness of the importance of screening strategies, but it may also be related to the high specialization of the participating cardiologists/electrophysiologists who treat patients already studied in earlier care-level settings.^5^

Although current guidelines^1^ require a single-lead ECG recording ≥30 s or 12-lead ECG to establish a definitive AF diagnosis, one-third of the physicians trust non–ECG-based algorithms. This fact could imply an overdiagnosis of AF.^6^

### Comorbidities/lifestyle programmes

A novel aspect of the 2020 guidelines is that the identification and management of risk factors and comorbidities becomes a pillar of AF treatment (Class I recommendation).^1^ Most of the participants consider the management of comorbidities and unhealthy lifestyle habits relevant; however, less than half has access to specific programmes and <10% has access to a comprehensive approach. This fact demonstrates a lack of health resources oriented to preventive strategies, hampering the implementation of recommendations that have demonstrated to have a great impact on the treatment and natural history of the disease.^7,8^

Interestingly, there is an evident gradient of accessibility in Europe from south to north and from west to east. These differences are also observed when considering the type of health care system. Those countries with Beveridge systems have access to such programmes in 54% of the cases while those based on Bismarck systems in 28%.

### Stroke prevention—anticoagulation and LAA closure

Most of the responders follow the general recommendations for anticoagulation.^1^ However, greater discrepancies were found in the anticoagulation approach for patients at high risk for bleeding. Despite the proven benefit of anticoagulation in almost all groups except for patients with advanced renal failure,^9–11^ responders generally showed a tendency towards a conservative management. Notably, liver disease patients were those more frequently consulted, although no observational study shows a significant increase in bleeding with NOACs that contraindicate their anticoagulation.^9^

After intracranial or gastrointestinal bleeding, many recommend the use of atrial appendage closure devices. This antithrombotic strategy has gained popularity in recent years compared to the results of a previous EHRA survey addressing stroke prevention approaches after intracranial haemorrhage.^12^ Guidelines only indicate LAA occlusion in cases of irreversible causes of bleeding or non-modifiable risk factors (Class IIb).^1^ Ongoing randomized clinical trials such as CHAMPION-AF (NCT 04394546) and CATALYST (NCT04226547) will provide more information in this field.

Surprisingly, there are still a small percentage of physicians who continue prescribing antiplatelet drugs in high-risk AF patients despite their proven inefficacy in stroke prevention and bleeding risk.^13^

#### Post-operative atrial fibrillation after non-cardiac surgery

Long-term OAC therapy to prevent thrombo-embolic events in this setting is a matter of great debate.^14^ The survey shows a great heterogeneity in clinical practice in this regard. There is an ongoing randomized clinical trial (ASPIRE AF; NTC03968393) currently trying to answer this question.

#### Atrial fibrillation episodes detected by cardiac implantable devices

The criteria to consider AHREs clinically relevant are controversial, and, as a result, it is not clear when to indicate long-term OAC.^15,16^ A recently published randomized study has failed in providing enough evidence supporting the use of NOAC in short-lasting AHRE of >6 min.^17^ A recent large retrospective cohort study showed large practice variations in OAC initiation for this type of patients.^18^ This survey has found similar variability.

### Arrhythmia management

Rhythm control is the preferred strategy in most AF phenotypes, with the lowest rates for persistent AF with risk for recurrence (72%) and asymptomatic paroxysmal AF (77%). In the case of asymptomatic AF, ablation is considered as first-line therapy for almost half of the physicians. Notably, ablation is the first-line therapy for more than half of the participants in persistent AF and in 10% of the participants for permanent AF. In accordance with the guidelines, none of these scenarios are recommended to perform an ablation as a first-line treatment.^1^ Compared to a recent EHRA survey, AF ablation has become a more widely indicated procedure across the spectrum of AF patients.^19^ For example, in patients with symptomatic paroxysmal AF, ablation as first-line treatment has increased from 42 to 69% in our study; and in the case of persistent AF, ablation has increased from a 7% as a routine first-line approach to a >50%. These observations may have two explanations: on the one hand, a belief that early rhythm control may halt the progression of the arrhythmogenic substrate^20,21^; on the other hand, technical advances have made pulmonary vein ablation a routine procedure with a very low percentage of severe complications, especially at high-volume centres.^22^

The ablation protocol contemplates further ablation targets beyond PVI in up to 32% of persistent AF patients. There is no evidence for this approach, which can be deleterious in patients with more vulnerable atria.^23^

Cavo-tricuspid isthmus ablation during the same procedure of AF ablation is broadly performed, reaching 80% if typical flutter is induced or clinically documented. However, according to the current guidelines, it is a Class IIb recommendation.^1^

Regarding the use of AADs, the discrepancy between the support attributed to dronedarone by the guidelines and its usage clearly stands out. This is a drug with a Class IA recommendation for long-term rhythm control in AF patients with preserved to mildly impaired LV function.^1^ However, more than half of the participants barely use it as first-choice drug, and its use is relegated to cases of thyroid disorders or failure of other drugs. The possible justifications are its lower efficacy with respect to amiodarone and its contraindication in multiple frequent scenarios in AF.^24^ Likewise, potential liver failure, which requires close laboratory monitoring, is cumbersome.

Vernakalant has a Class IA recommendation for pharmacological cardioversion of new-onset AF in cases with no recent acute coronary syndrome or severe heart failure.^1^ This drug has higher effectivity than amiodarone or flecainide, a fast onset of action, and a relative selectivity for atrial myocardium.^25^ Its elevated costs, the contraindication in case of severe heart failure or recent acute coronary syndrome, and the incorporation of a new unknown drug in the therapeutic arsenal of the physicians may limit its routine use as the survey shows.

### Lack of accordance with guidelines

As shown in the results of this survey, there is a lack of accordance with current guidelines in many of the diagnostic and therapeutic fields. Several hypotheses may explain this fact: (i) economical barriers may hinder the implementation of strategies requiring specific programmes and personnel or more expensive treatments; (ii) the relative scepticism of physicians about guidelines developed in recent years due to the level of scientific evidence and uncertainty supporting some of the recommendations^26^; (iii) guideline redundancy in the last years (the number of European guidelines published each year has doubled since 2018); and (iv) lack of adequate continuous medical education.

### Limitations

The results of this survey must be extrapolated to the whole EHRA community with caution, since 40% of the answers came from Italy and Spain, resulting in an overrepresentation of Mediterranean countries, and 18% from Germany. Nevertheless, we think it is not a noteworthy limitation since Mediterranean participants are mainly integrated in the Beveridge health care system whereas German participants exemplify the Bismarck health care system, providing a general idea of the trends present in the EHRA community.

Since 91% of the participants perform AF ablations in their daily practice, the conclusions of the study are mainly extrapolable to the management of interventional electrophysiologists.

Additionally, the voluntary nature of the survey favours selection bias and raises questions whether these results represent a realistic reflection of the current practice. Potential knowledge gaps regarding the guideline content may merit reinforcing some areas of continual medical education. However, a more thorough assessment and understanding of the reasons underlying low adoption of some recommendations should be pursued.

## Conclusions

The results of this survey show marked heterogeneity in the management of AF patients in the EHRA community and relatively low accordance with the latest ESC guidelines. These findings mainly concern the most controversial issues, such as those related to AF screening and interpretation of non–ECG-based monitoring tools, management of comorbidities, pharmacological treatment, and ablation strategy. Different geographical and economical environments seem to impact the implementation of guideline recommendations. An effort is required by scientific societies and other stakeholders, going beyond guideline development, to try to homogenize clinical practice and increase the use of best evidence practice medicine to obtain optimal results in a generalized manner.

## Supplementary Material

euae012_Supplementary_Data

## Data Availability

The data that support the findings of this study are available from the corresponding author upon reasonable request.
