# Supplementary material for: Current management of atrial fibrillation in routine practice according to the last ESC guidelines: an EHRA physician survey—how are we dealing with controversial approaches?
Source: Europace. 2024 Jan 16;26(2):euae012. doi: 10.1093/europace/euae012 (PMC10869216; doi:10.1093/europace/euae012)
Supplement: euae012_Supplementary_Data [file euae012_supplementary_data.pdf]

Supplementary Table. Distribution of country of origin of participants.

**Country**	**Number of participants**	**Percentage**
Argentina	4	2.2
Austria	2	1.1
Belgium	1	0.5
Brazil	1	0.5
Bulgaria	1	0.5
Canada	2	1.1
Costa Rica	1	0.5
Croatia	3	1.6
Denmark	1	0.5
Ecuador	1	0.5
Egypt	2	1.1
France	3	1.6
Germany	33	18.1
Greece	5	2.7
Hong Kong	1	0.5
Hungary	1	0.5
India	1	0.5
Ireland	1	0.5
Israel	3	1.6
Italy	13	7.1
Lebanon	1	0.5
Malta	1	0.5
Mexico	1	0.5
Netherlands	4	2.2
Peru	1	0.5
Philippines	1	0.5
Poland	4	2.2
Romania	1	0.5
Serbia	1	0.5
Slovenia	2	1.1
Spain	60	33
Sweden	2	1.1
Switzerland	1	0.5
Turkey	1	0.5
United Kingdom	7	3.8
Uruguay	1	0.5
Unknown	12	6.6

SURVEY

Current management of AF and adherence to 2020 Guidelines:
Physicians-based survey

The aims of this survey are :

- To describe the current clinical practice in AF within the EHRA/ESC community
- To assess adherence to the recent ESC Guidelines published in 2020,
- To focus on the most controversial management points.
- Ultimately, to detect discrepancies between the recommendations and current treatment management / patterns.

[Add in the disclaimer]

Overall, do you treat or follow-up AF patients?
Yes (continue)
No (exclude from survey)

1. **What country do you work in?**

2. **How many years of experience do you have in the management of AF patients?**
   "Slide with a year's range" 0 to +35

3. **What type of institution do you work in?**
   a. University Hospital
   b. Non-University Hospital
   c. Specialised Cardiology Centre
   d. Private centre
   e. Other. Please specify.

4. **Which of the following services can be found in your institution?** (multiple answers possible)
   a. Outpatient cardiology
   b. Cardiology ward
   c. EP department
   d. Anticoagulation department
   e. AF ablation
   f. Pacemaker/ICDs implantation and follow-up
   g. LAA closure
   h. Cardiac surgery

5. **How many AF patients do you usually treat in a regular month?**
    a. AF ablation: "Slider with range: 0 to >50
    b. LAA closure: "Slider with range: 0 to >50
    c. Outpatient follow-up: "Slider with range: 0 to >200

6. **Are you performing follow-ups of AF patients?**
    a. Never
    b. Only short time after ablation
    c. Long time after ablation
    d. Medical management (no matter ablation status)

7. **Are you the main cardiologist who perform the holistic diagnosis/management of the AF in your patients?**
    a. Yes/frequently.
    b. No/unfrequently (I am more involved in procedures)

8. **How often do you perform opportunistic or systematic AF screening in high-risk patients (>65y, arterial hypertension, OSAS…)?**
    a. No/rarely
    b. Not by myself, but opportunistic screening is performed in my reference area
    c. Occasionally
    d. Yes/frequently

9. **Which tools are used for AF screening in your reference area? (multiple answers possible)**
    a. By pulse taking/auscultation in the consultation
    b. By rhythm strip/12-leads ECG in the consultation
    c. By Holter monitoring
    d. I recommend the patient to use wearables like watches or smartphones
    e. Others. Please specify
    f. N/A

10. Do you consider automatic AF diagnostics (irregular rhythm notifications) from wearables/cardiac implantable devices in your diagnostic work-up?

a. Rarely, I do not trust them/ I do not check them regularly
b. Only if tracings/EGM of the device are available and well recognizable
c. Only after confirming with a standard Holter monitoring or 12-lead ECG
d. Frequently, also episodes without available tracings (based on photopletysmogram or AHRE detection by implantable devices)
e. N/A

11 . Do you have access to dedicated resources/programmes for the management of unhealthy lifestyles and comorbidities for your AF patients?

a. No, I am not involved in that aspect of AF management
b. No, I do not have access to those. I do it by myself
c. Yes, I have access to a specific programme for the diagnosis and management of comorbidities and lifestyle modification

If you answered yes, what resources do you have?

a. Educational programmes designed by nurses
b. Resources from nutritionists
c. Resources from endocrinologists
d. Exercise programmes
e. Sleep Unit (OSAS screening/management)
f. Other. Please, specify

12. Do you consider postponing your decision about the indication of ablation based on the control of comorbidities/unhealthy lifestyles?

a. Rarely
b. Occasionally
c. Frequently
d. N/A

13. Which is your regular strategy for thrombo-embolic prevention in low-risk patients (CHADS-VASc =1 in men/=2 in women)?

a. No antithrombotic/antiplatelet treatment
b. I recommend starting antiplatelet treatment
c. I recommend starting anticoagulation
d. I discuss the pros- and cons- with the patient
e. My recommendation is based on the relative weight of each risk factor

14. **What type of anticoagulation is mostly prescribed to your AF patients?**
    a. Anti-vitamin K
    b. NOAC
    c. NOAC only if TTR<70%

15. **What is your general strategy for the thrombo-embolic prevention in these high-risk groups?**

	ACO	Antiplatelet	LAA closure	Discuss with patient	No treatment
HASBLED score >=3					
Extreme ages (>90y)/dementia					
Advanced kidney disease					
Liver impairment/cirrhosis					
Cancer					
After cerebral haemorrhage					
Gastrointestinal bleeding					

16. **What is your thrombo-embolic prevention strategy in self-limited postoperative AF after non-cardiac surgery in patients with elevated CHA2DS2-VASc?**
    a. I rarely recommend anticoagulation
    b. I frequently recommend anticoagulation, but only short-term (3 months)
    c. I frequently recommend long-term anticoagulation
    d. I discuss pros and cons with the patient

17. **When do you recommend anticoagulation in AF patients with AHRE registered in cardiac implantable devices and elevated CHAD-VASc?**
    a. Never/rarely
    b. Only if episodes last more than 5-6 minutes, but not if episodes last a few seconds-minutes
    c. Only if episodes last more than 24 hours
    d. Only if AF burden is important or a progression in the episode’s duration is observed (no matter single episode duration)
    e. Always if CHAD-VASc is very high (no matter the duration or burden)
    f. Always (no matter the duration or burden)
    g. N/A

18. Which is your first-choice strategy for the rhythm management based on the following AF phenotypes?

Asymptomatic parox AF	Symptomatic parox AF	Persistent AF wo* recurrence risk	Persistent AF w* recurrence risk	Long-standing persistent AF/permanent	AF + reduced ejection fraction/TIC**
Rhythm control	Rhythm control	Rhythm control	Rhythm control	Rhythm control	Rhythm control
Rate control	Rate control	Rate control	Rate control	Rate control	Rate control

*Risk of AF recurrence: LA enlargement/atrial fibrosis; **Tachycardia-induced cardiomyopathy

19. **Which drugs do you use in the following settings?** (click each option if you use the drug often in your regular management of the condition, and do not click it if you don't use it or you use it only occasionally):

	Asymptomatic paroxysmal AF	Symptomatic paroxysmal AF	Persistent AF wo* recurrence risk	Persistent AF w* recurrence risk	Long-standing persistent AF/permanent AF	AF + reduced ejection fraction/TIC**
Ia (bepridil, cibenzoline, disopyramide, procainamide, quinidine)						
Ic (flecainide, propafenone)						
B-blockers						
Amiodarone						
Dronedarone						
Sotalol						
Calcium-antagonists						
Digoxin						

*Risk of AF recurrence: LA enlargement/atrial fibrosis; **Tachycardia-induced cardiomyopathy

20. **Regarding dronedarone. When do you use it?** (multiple responses possible)

a. Never /Rarely
b. For those patients not tolerating any other drug
c. When amiodarone use is not recommended due to thyroid problems
d. When other drugs fail
e. As a first line medical therapy

21. When do you consider AF ablation in these patients?

	Asymptomatic parox AF	Symptomatic parox AF	Persistent AF wo* recurrence risk	Persistent AF w* recurrence risk	Long-standing persistent AF/permanent AF	AF + reduced ejection fraction/TIC**
First option						
Second option after drug/***ECV failure						
Never						

*Risk of AF recurrence: LA enlargement/atrial fibrosis; **Tachycardia-induced cardiomyopathy; ***ECV = electric cardioversion

22. What is your experience with vernakalant for pharmacological cardioversion?

a. I have no access to it in my institution
b. I have access to it in my institution, but I never/rarely use it
c. I use it frequently
d. N/A

23. Is your decision on rhythm-control vs. rate-control strategy influenced by the following factors?

	Never/rarely	Often	Always
**Age**			
**AF phenotype**			
**LA remodelling**			
**Comorbidities**			
**Symptoms**			
**Patient's decision**			

Others: please specify

24. Which is the maximum patient's age that you consider for AF ablation?

a. "Slide with a year's range" 60-80
b. I do not consider age as a limitation for AF ablation

25. Which is your regular ablation approach for paroxysmal AF?

a. PVI only
b. PVI + additional ablation lesions (lines, low-voltage, rotors…)
c. Surgical ablation
d. N/A

26. Which is your regular ablation approach for persisting AF?

   a. PVI only
   b. PVI + additional ablation lesions (lines, low-voltage, rotors…)
   c. Surgical ablation
   d. N/A

27. **To whom do you offer redo AF ablation**? (multiple responses possible)

   a. Rarely / never
   b. Only if symptoms improved after the first procedure
   c. To all paroxysmal AF
   d. To PerAF without LA fibrosis, severe dilatation, or significant electrical disease at the index procedure
   e. Only if left flutter appears during follow-up
   f. To most/all patients

28. How often do you perform CTI ablation during the same procedure as AF ablation?

   a. Never/rarely
   b. Only if the patient has a history of previously documented typical flutter on an ECG
   c. Only if the patient has a documented typical flutter or if it is induced during the procedure
   d. Always/often
   e. N/A

29. Do you have access to surgical AF ablation in your institution?

   a. No, I do not have access to it
   b. Yes, it is performed as concomitant cardiac surgery
   c. Yes, as a stand-alone surgery or hybrid procedure

30. How often do you use the pace and ablate approach (AV node ablation + pacemaker)? (multiple responses possible)

   a. Never/rarely
   b. For those patients probably not having any benefit from ablation (before trying ablation)
   c. As a last resource (after ablation) when the patient is very symptomatic or the heart rate difficult to control
   d. When ablation fails (no matter symptoms or heart rate)
   e. Frequently (no matter ablation status)
